# Environmentally sustainable mining in quarries to reduce waste production and loss of resources using the developed optimization algorithm

**DOI:** 10.1038/s41598-023-49633-w

**Published:** 2023-12-13

**Authors:** Mohammad Hossein Jalalian, Raheb Bagherpour, Mehrbod Khoshouei

**Affiliations:** Department of Mining Engineering, Isfahan University of Technology, Isfahan, 8415683111 Iran

**Keywords:** Environmental impact, Sustainability, Environmental sciences, Environmental social sciences, Engineering, Geology, Tectonics

## Abstract

The study of natural resources in the earth sciences focuses on the sustainable management of valuable materials like dimension stones. the quarrying of dimension stones is associated with environmental challenges such as significant amounts of waste production, and resource loss, mainly caused by discontinuities and fractures in the rock mass. Quarry optimization requires an optimal cutting pattern to increase the production of larger blocks while minimizing parameters that affect operational costs such as energy consumption. The algorithms used in the quarrying only focus on the number of blocks extracted, ignoring other factors such as energy consumption in the cutting of blocks. To address this issue, a new algorithm was developed in this study. The algorithm aims to optimize the quarrying process by analyzing the impact of discontinuities on waste production and cutting surfaces. It then provides an optimal cutting pattern for the quarry face based on the optimal value of these parameters. As a result, the use of this algorithm can serve as an efficient and valuable tool in dimension stone quarries. By implementing this algorithm, production costs, energy, and water consumption, cutting tools consumption, and waste production can be significantly reduced, leading to increased quarry profitability and decreased environmental problems.

## Introduction

**Figure 1:**
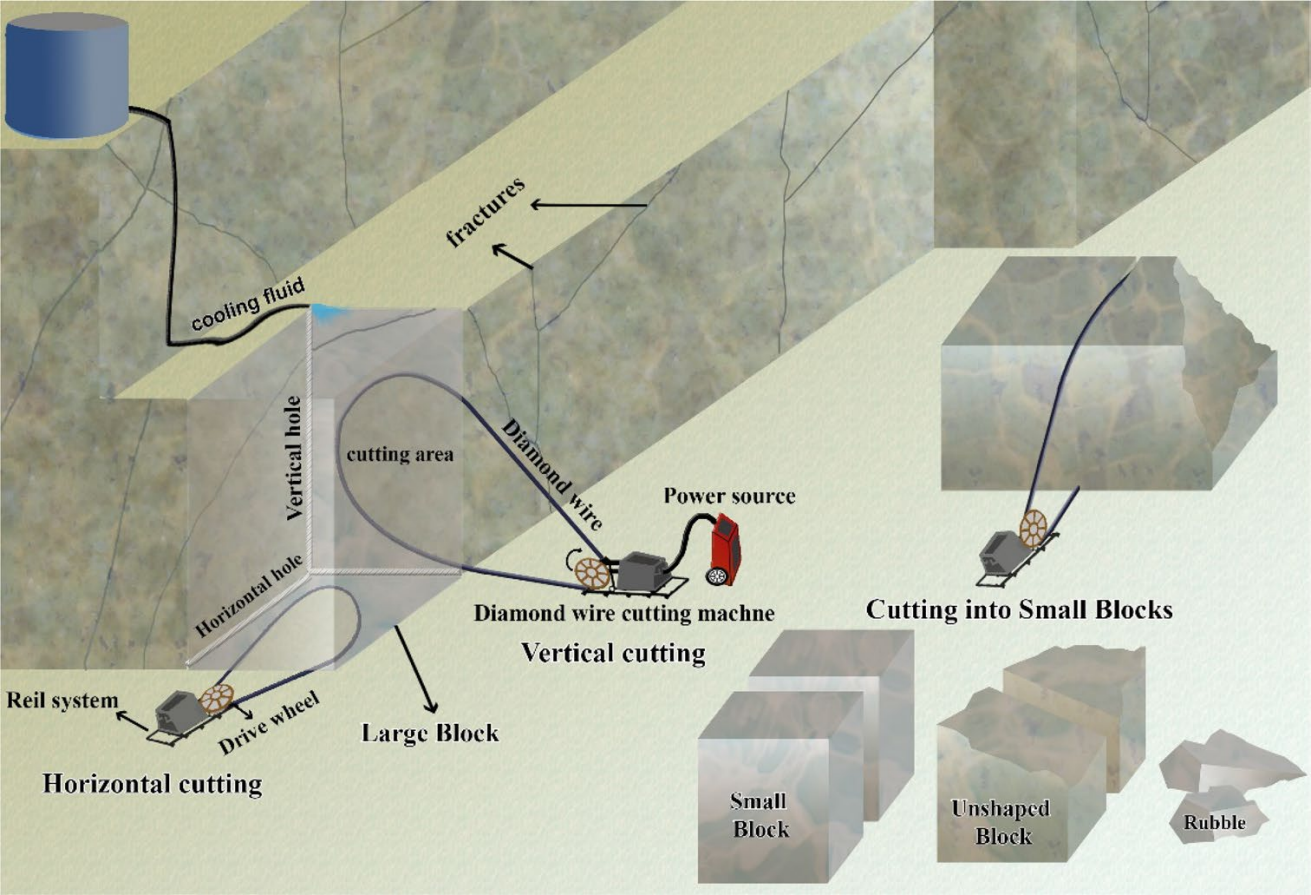
A view of dimension stones quarry stage^7^.

Dimension stones are natural stones that have standard dimensions and shapes, and are used in various construction fields such as building facades, interior parts of buildings, structures, sculptures, paving, and more^1^. Dimension stones have a distinct advantage over other building materials due to their appearance and decorative applications, which are determined by their color combination, uniform texture, and lack of discontinuity. They can also be classified based on these factors^2^. In recent years, due to the increase in construction, which is influenced by the expansion of urbanization, the dimension stones industry has become an industry with significant and increasing annual production and an industry with high economic potential. Besides, the level of profitability in this industry depends on the efficiency of the production cycle. The dimension stones production cycle consists of three main stages: exploration, quarry, and processing. After exploring the dimension stones deposit, the quarry is prepared, and large blocks are cut and extracted. Cutting the block from the quarry face requires non-destructive methods that minimize the damage to the block and increase efficiency. The most common methods for dimension stone quarrying are diamond wire cutting, chainsaw, cutting disc, flame jet, blasting methods, etc.^3^. The most common method of cutting blocks in dimension stone quarries is the diamond wire cutting method, which has been used since 1985 and is still used in 90% of dimension stone quarries today^4,5^. The advantages of this method include high cutting speed, more production efficiency, and increased block production^6^. The next step, cutting large blocks into smaller blocks to facilitate transportation is performed, and these blocks are transferred to the processing plant. Figure 1 shows a view of the dimension stones quarry stage.

**Figure 2:**
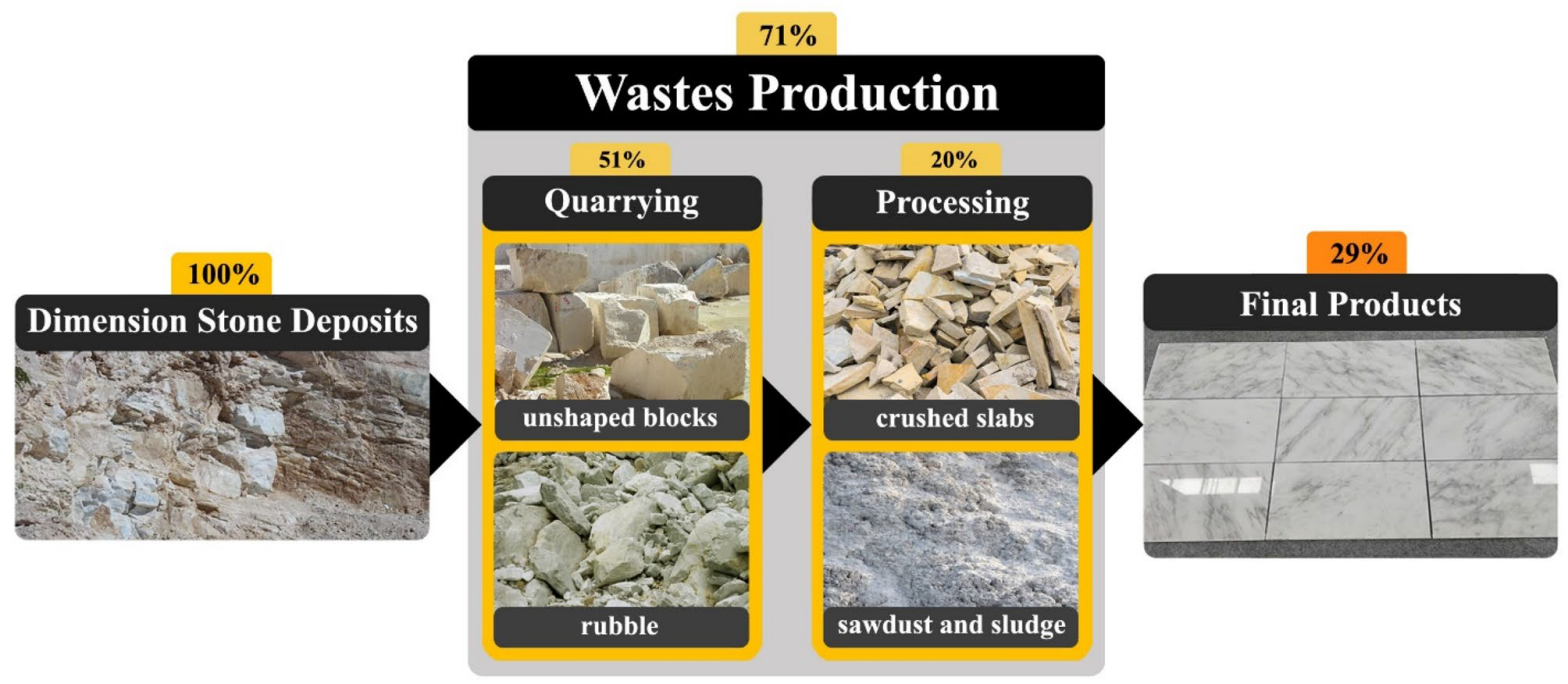
Products and wastes in different stages of the dimension stones production cycle.

The blocks that are transported to the processing plant are sorted based on their dimensions and specifications. They are then cut into final products based on their intended use, which are typically slabs or tiles. Depending on the intended market, the surfaces of these products are smoothed and polished to transform them into the desired finished product^8^. Factors affecting the efficiency of the dimension stones production cycle include the enormous amounts of waste production, resource loss, and the amount of energy and consumables. According to the data published in 2021 by Montani, from the total 318 million tons of gross quarrying from dimension stone quarries, during the quarry stage, about 163 million tons, or in other words, about 51% of total gross quarrying has become quarrying waste. The most important factors affecting the waste generated in the quarry stage are the geological conditions of the deposit, including discontinuities and fractures in the rock mass, which mainly lead to waste products such as unshaped blocks and rubbles^9^. As shown in Fig. 2, in the continuation of the production cycle, about 49% of the remaining raw production is transferred to the dimension stones processing plant and its amount is about 155 million tons, nearly 63.5 million tons or 41% (about 20% of the total gross quarrying) is converted to the waste during the processing stage, which mainly includes waste such as crushed slabs, sludge and sawdust^10,11^. According to the above contents, it can be concluded that of the total gross quarrying in the dimension stone production cycle, only 91.5 million tons, or 29% was marketed as processed products, and about 71% of it was lost as waste production.

While quarries are potential economic locations, further attention is needed to guarantee a cost-effective and environmentally efficient system for waste management in these places^12^. To improve the production efficiency of dimension stones, it is important to minimize the amount of waste and resource loss with optimization methods and technologies. In case it is not possible to reduce waste and loss, recycling or reuse methods should be implemented in the future^7^. Numerous studies have been conducted in recent years to enhance the production efficiency of dimension stones. These studies can be broadly categorized into two stages—quarrying and processing. The quarry stage focuses on identifying and modeling discontinuities, determining the specifications and geometry of in-situ blocks in the rock mass, and using block quarry optimization algorithms. Meanwhile, methods to improve efficiency in the processing stage involve the use of optimal devices and technologies, as well as recycling waste production. Table 1 summarizes some of the methods used in these studies.

**Table 1:** Studies on increasing the production efficiency of dimension stones.

Author(s)	Year	Method	Results
Grandjean and Gourry^13^	1996	Detection and modeling of the fractures using GPR	Determining the number of stone blocks and areas without fractures
Porsani et al.^14^	2006	Detection of discontinuities using GPR	Identifying formed blocks, reducing the number of explosives consumed, and reducing production costs
Kadioglu^15^	2008	Detection and modeling of the discontinuities with GPR	Determining the characteristics of discontinuities and detecting the texture of different layers
Ülker and Turanboy^16^	2009	Detection of in-situ blocks using genetic algorithm	Determining the cube blocks with the maximum volume that can be installed in specified natural blocks, determining the total recovery rate
Mosch et al.^17^	2011	Detection of the blocks without fracture and their number using the *3D-Block Expert* application	Determining the optimal cutting direction
Álvarez-Fernández et al.^18^	2012	Detection of the best cutting angle of slabs using a computational algorithm	Determining the optimal slab cutting pattern, producing slabs with the maximum possible surface
Gazi et al.^19^	2012	Evaluation of the energy condition and environmental performance of different dimension stones processing stages	Determining the energy consumption in different dimension stones processing stages, determining the importance of optimization in each stage
Fernández-de Arriba et al.^20^	2013	Predicting and optimizing the cutting the commercial-sized blocks using the *CUT ROCK* application	Determining the optimal cutting direction, increasing efficiency
Mendoza et al.^21^	2014	Assessing the performance of different saw technologies, Investigation of rainwater harvesting potential	Determining the optimal saw technology in water consumption, energy consumption, consumables materials, and efficiency
Yarahmadi et al.^22^	2014	Detection of the geometry of rock mass blocks using a 2D algorithm	Determining the geometry of the 2D blocks and their number
Careddu et al.^23^	2014	Investigating the recovery of sawdust from marble processing plants	Identification of the usability of sawdust in the manufacture of industrial products such as paper, paint, and rubber
Rey et al. ^24^	2015	Detection of discontinuities using GPR	Identification and separation of different rock layers and identification of the small fractures in the rock mass
Elkarmoty et al.^25^	2017	Detection and modeling the discontinuities using GPR	Identification areas with fewer fractures
Elkarmoty et al.^26^	2017	Detection and modeling the discontinuities using GPR	Determining the characteristics of discontinuities and their number
Elkarmoty et al.^27^	2017	Detection of the discontinuities and fractures using GPR	Detection of non-extractable quarry faces due to many fractures, determining an area with the lowest fracture number
Yarahmadi et al.^28^	2018	Detection of the geometry of rock mass blocks using the *3D-Quarry Optimizer* application	Determining the optimum quarrying direction, determining the interval between the vertical cutting, increasing production efficiency
Elkarmoty et al.^29^	2018	Detection and modeling the discontinuities using GPR	Determining the characteristics of discontinuities
Marras and Careddu^30^	2018	recycling of marble waste in tire mixture production	Identification of the way to use marble sludge as filler in high-added-value products
Yurdakul^31^	2020	Investigation of wastes produced in dimension stones processing plants	Determining the amount of waste production, classification of wastes by size and quality
Elkarmoty et al.^32^	2020	Optimizing the number of non-fracture blocks using the *BlockCutOpt* application	Increase the number of non-fracture blocks, increase the production efficiency
Elkarmoty et al.^33^	2020	optimizing the cutting of slabs from dimension stones blocks using the *SlabCutOpt* application	Increase recovery ratio and revenue, decrease waste production
Adrián Riquelme et al.^34^	2022	Control of natural fractures in historical quarries via 3D point cloud analysis	The optimum orientation of the quarry face for minimizing efforts and rock waste

According to Fig. 2, the quarry stage of the dimension stones production cycle is responsible for most of the waste production and resource loss. The primary reason for waste production in this stage is the presence of discontinuities and fractures in the rock mass, which reduce the size of extracted blocks and increase waste generation. In recent years, researchers have shown interest in optimizing dimension stone quarries to increase efficiency and reduce waste production. Table 1 lists some of the methods used by these researchers, which have proven to be effective in increasing production efficiency and reducing waste. However, it is notable that these studies have not paid much attention to other parameters, such as energy consumption, that could also improve the efficiency of dimension stone quarries.

Many factors impact the economic and environmental optimization of dimension stone quarrying, including operational costs, waste production, water usage, energy consumption, and cutting tool consumption. However, the optimization methods used in dimension stone quarries have only paid attention to the amount of production of economic blocks and the amount of waste, and other important parameters have not been paid attention to. For example, in cutting blocks from the quarry face, in addition to the dimensions of the blocks, the number of surfaces that must be cut to extract the desired blocks from the quarry face is also very important. Cutting surfaces affect the amount of energy, water, and cutting tool consumption. Besides, increasing the amount of waste production and resource loss causes the energy and consumables that should be used to produce the product, used to waste production, and increases the amount of energy and consumables per product production. Increasing the amount of waste production and subsequently reducing the production of the product, along with increasing the energy consumption and consumables, increases the production cost and significantly reduces production efficiency. All these parameters affect the economic and environmental optimization of dimension stone quarries and should be seen simultaneously. In other words, paying attention simultaneously to all effective parameters in increasing quarry efficiency can show more effective results. Optimization of the dimension stones quarry stage requires that while examining the optimal cutting pattern to increase the production of blocks with maximum dimensions, consider minimizing the parameters such as cutting surfaces, energy consumption, and consumables. Accordingly, the optimization results can be more comprehensive and increase production efficiency as much as possible.

In this paper, an optimization algorithm is developed to simultaneously optimize the economic and environmental factors of dimension stone quarries. After receiving the information on discontinuities and fractures of the rock mass, as well as considering the mining limitations, this algorithm provides the optimal cutting pattern of the quarry. The main difference between the presented optimization algorithm and previous algorithms is that it considers parameters such as the cutting surfaces of the blocks, in addition to the amount of production of economic blocks and the amount of waste. Attention to this parameter has an effective impact on energy consumption, water, and consumables. The details of the developed optimization algorithm, along with its results, are presented in the next sections.

## Methodology

**Figure 3:**
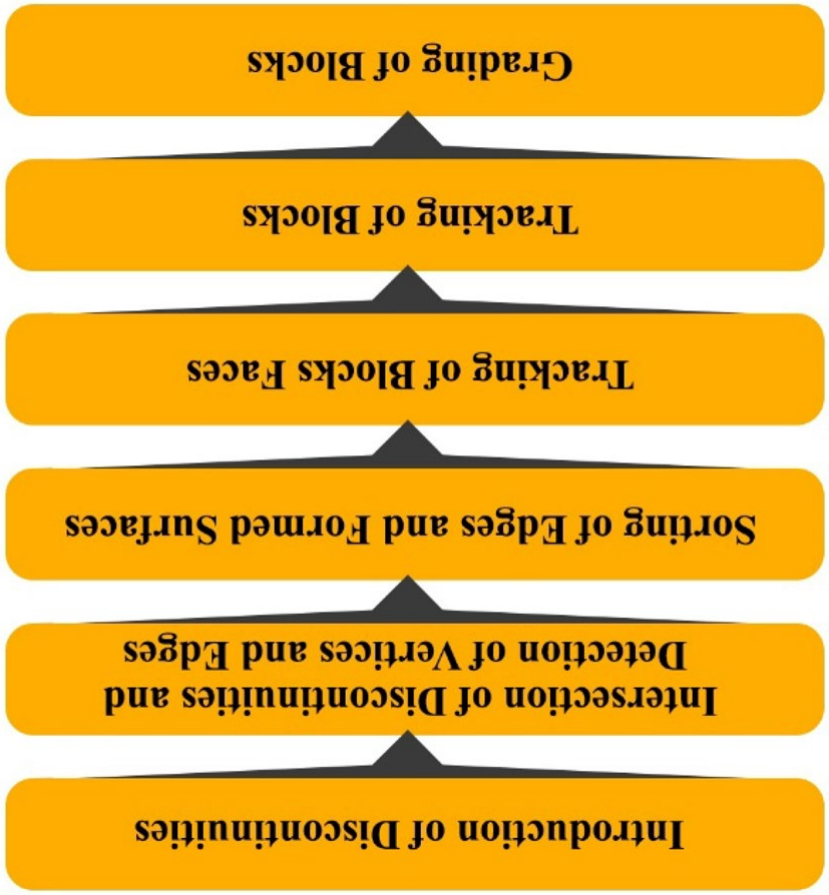
Steps for identifying and grading blocks in the base algorithm.

Several quarry optimization algorithms have been developed based on block geometry modeling. However, these algorithms mainly focus on the amount of production of economic blocks and neglect other parameters such as the energy used to cut the blocks from the quarry face, which can affect production costs. Therefore, it is necessary to develop an algorithm that can simultaneously consider the main parameters affecting the economic and environmental optimization of dimension stones quarrying. Among the previous algorithms, the algorithm developed by Yarahmadi et al.^28^ in 2018, was selected as the base algorithm due to its ability to model complete and incomplete discontinuities, determine the geometry of blocks, 3D modeling, and grading of blocks. The method of this algorithm is that first, the discontinuities are considered as planes and are given intersections. Edges and vertices are then identified and sorted. The block's faces are then tracked, and using these faces, the blocks are tracked, and finally, the identified blocks are graded based on their volume and shape. The larger the volume of the tracked block and the more similar its shape to a rectangular cube block with index dimensions, the higher the class of the block. The steps of this algorithm are shown in Fig. 3.

Based on the given explanations, it is necessary to make changes in the direction of achieving the set goals when using the base algorithm and to update it. This implies focusing on all the parameters that affect the economic and environmental optimization of dimension stones quarrying. In the following section, the development of a new algorithm that aligns with the set goals will be explained.

### Developed algorithm

**Figure 4:**
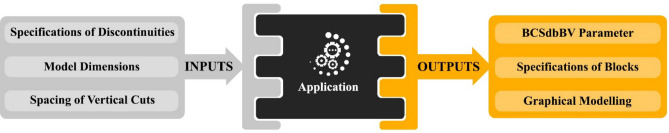
General process of the developed algorithm.

Different algorithms require implementation and conversion into computer programs. Programming languages such as MATLAB, C++, and Pascal are used to prepare computer programs. Each software has its advantages and disadvantages and is used based on the user's needs. in this study, MATLAB programming language was used as it is based on matrices and has excellent graphical capabilities. A newly developed algorithm was programmed using MATLAB. The general structure of the new algorithm is shown in Fig. 4.

Figure 4 shows how the new algorithm receives input data, which includes the discontinuity profile, model dimensions, and spacing of vertical cuts on the quarry face. The algorithm's first input is the characteristics of discontinuities, which include four main items- the type of discontinuity (complete or incomplete), the slope, the direction of slope, as well as a point of the plane of discontinuity (X, Y, and Z); that is given as input to the algorithm. The second input of the algorithm is the dimensions of the model. For example, in this study, the target model is a dimension stone quarry face, and the dimensions of the face, such as length, cutting depth, and height, are given as input to the algorithm. The third input of the algorithm is the distance of the vertical cuts in the quarry face. Vertical cuts are made to separate large blocks from the face, which are usually fixed (1.8 to 2 m) in dimension stone quarries.

After receiving these inputs, the developed algorithm first considers the dimensions of the model (length, depth, and height) and creates a 3D space of a rectangular cube under the title "model space". The algorithm then models the discontinuities according to their specifications in the form of planes in the "model space". According to their spacing, vertical cuts are considered vertical planes and are modeled in "model space". Finally, all the modeled planes in the 3D space are intersected and lead to the formation of in-situ blocks. The algorithm also identifies the number of cuts required to separate large blocks (rectangular cubic blocks extracted from the quarry face of dimension stone quarries) from the quarry face. It should be noted that the amount of cutting has a direct relationship with energy, water, and cutting tool consumption. After running the algorithm, it finally provides the specifications of the tracked blocks and 3D graphic modeling of the quarry face as the primary output. The algorithm also provides *Block Cutting Surfaces divided by Block Value* (BCSdbBV) parameter as the main output and the main optimization parameter. In BCSdbBV calculation, two main parameters are effective, including the block value and the block cutting surfaces. The calculation of the BCSdbBV parameter is explained below.

#### Block Cutting Surfaces (BCS)

In dimension stone quarrying, blocks are separated from the quarry face by cutting their faces. To separate a rectangular cube block from the quarry face, four faces need to be cut. These four faces include two side faces, the back face, and the bottom face of the block. In this study, the total area that needs to be cut to separate the block from the quarry face is referred to as the BCS, measured in square meters. The value of BCS can be calculated using Eq. (1).

1 $${\text{BCS}}=2{\text{SF}}+{\text{BF}}+{\text{UF}}$$

where BCS is the area of cutting faces to separate a block from the quarry face, SF is the area of the side face, BF is the area of the back face and UF is the area of the bottom face, whose unit is m^2^.

The BCS parameter represents the operating costs of quarrying, such as energy, water, and tool consumption. The higher the BCS value, the higher the energy and water consumption during the separation of the block from the quarry face, and the consumption of cutting tools. The increase in the consumption of energy, water, and cutting tools in the extraction of dimension stones leads to an increase in operating costs and, as a result, a decrease in production efficiency. According to the description, the BCS value calculation is the first parameter that is considered in the BCSdbBV calculation. In the following, the method of calculating the second effective parameter in the calculation of BCSdbBV, that is, the block value parameter, is explained.

#### Block Value (BV)

The value of a block extracted from a quarry is determined by its volume and shape, which are influenced by the discontinuities of the rock mass. This study measures the block's value based on its useful volume, which is calculated by multiplying the block's volume by its shape factor^28^. A block's value increases if its volume is larger and its shape is closer to that of a block with index dimensions (the ideal block for the target market). This increase in value leads to a reduction in waste production in dimension stone quarries, thus increasing production efficiency. Calculating the BV value is the second parameter considered in the BCSdbBV calculation. The following section describes the method of calculating the BCSdbBV parameter using the developed algorithm.

#### Block Cutting Surfaces divided by Block Value (BCSdbBV)

According to the explanations and points expressed in the previous sections, the economic and environmental optimization of dimension stone quarrying require simultaneous attention to the following two items:

1. Paying attention to the parameters affecting operational costs (such as energy, water, and cutting tools consumption) can be achieved through investigation of the amount of BCS.
2. Paying attention to the amount of waste production (increasing the useful volume of extracted blocks) can be achieved through investigation of the amount of BV.

The parameter BCSdbBV is considered as the main goal of the developed algorithm. Equation (2) shows this parameter:

2 $${\text{BCSdbBV}}=\frac{{\text{BCS}}}{{\text{BV}}}$$

BCSdbBV indicates the amount of cutting surfaces per unit of valuable block. In other words, reducing the amount of cutting surfaces and increasing the value of the extracted block leads to a decrease in this parameter and consequently an increase in production efficiency. According to the points mentioned, the calculation of BCSdbBV has been selected as the main goal of the developed algorithm in this study.

### Implementation and evaluation of the developed algorithm

To show how to implement the developed algorithm, two hypothetical models named "Model 1" and "Model 2" were considered and the algorithm was evaluated on them. The dimensions of the hypothetical models were considered 2 m in the x direction (cutting depth of the quarry face), 20 m in the y direction (quarry face length), and 4.5 m in the z direction (quarry face height). The dimensions of the models are based on the common dimensions of quarry faces in dimension stone quarries. In these hypothetical models, each one contains several discontinuities with different characteristics, which were randomly selected among the discontinuities taken in the study of Yarahmadi et al.^28^. In the following, the characteristics of the discontinuities and the results obtained from the implementation of the algorithm on each model are presented.

#### Model 1

To evaluate the developed algorithm, a hypothetical quarry face named "Model 1" was considered. It included 7 discontinuities with different characteristics, which are shown in Table 2.

**Table 2:** Specifications of discontinuities in “Model 1”.

Joint number	Joint type	Dip (degree)	Dip direction (degree)	Coordinates of a point		
				X	Y	Z
1	Complete^a^	80	170	2	1	0
2	Complete	65	40	2	5	0
3	Complete	75	200	2	7	0
4	Complete	60	180	2	8.5	0
5	Complete	70	50	2	13.5	0
6	Complete	45	160	2	15	0
7	Complete	75	0	2	18	0

^a^A joint that has unlimited dimensions and is larger than the model dimensions.

**Figure 5:**
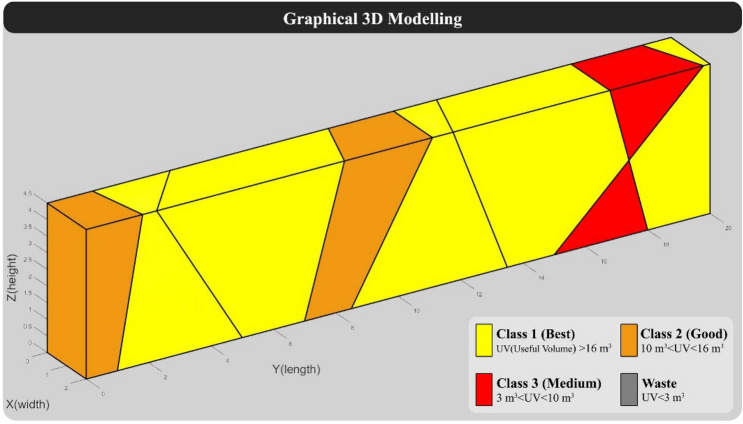
Graphical 3D modeling of “Model 1” without considering vertical cuts.

The dimensions of the large blocks in this model are considered 2 × 2 × 4.5 m (Common dimensions of large blocks in the quarry face). The direction of the quarry in this model was considered 270 degrees, and the spacing of vertical cuts in the quarry face was assumed to be constant and is considered 2 m according to traditional quarry methods (In traditional quarrying, the spacing between vertical cuts is 1.8 m to 2 m). In the block grading step, blocks with a useful volume of more than 16 m^3^ as class 1, between 10 and 16 m^3^ as class 2, between 3 to 10 m^3^ as class 3, and less than 3 m^3^ as waste are considered. Also, the value of class 2 and class 3 blocks are considered respectively 0.25 and 0.1 of the value of class 1 blocks, and the value of the waste block is considered equal to 0. It should be noted that the assumptions such as the dimensions of the quarry face, the dimensions of the large block, grading, and the value of blocks are considered based on the case study in Yarahmadi et al.^28^. According to the specifications of "Model 1", the programmed algorithm was implemented on it, and the results are displayed below. 3D modeling of "Model 1" without considering the vertical cuts of the quarry face is shown in Fig. 5.

**Figure 6:**
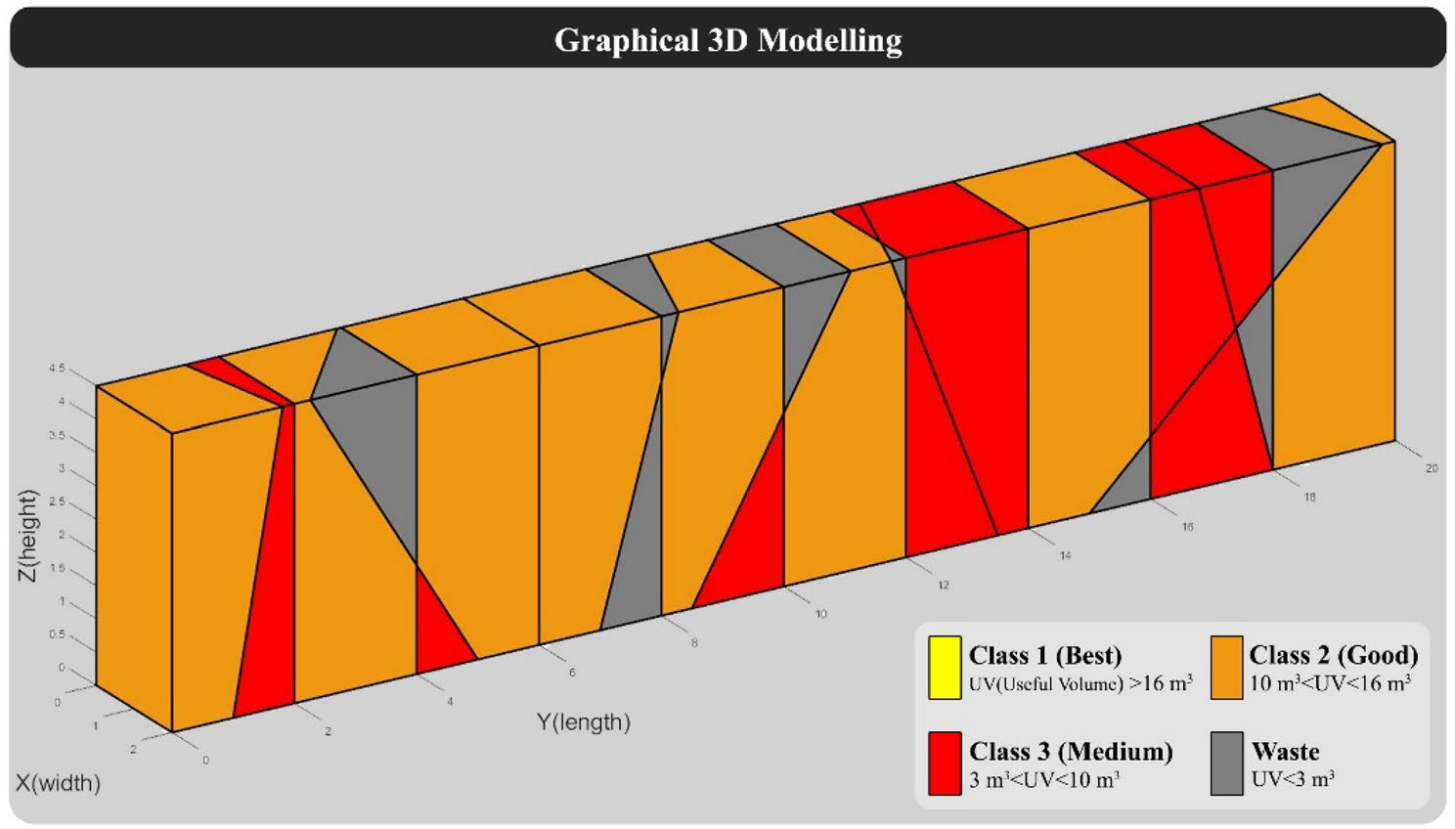
3D modeling of “Model 1” with a traditional cutting pattern.

For the hypothetical quarry face, the spacing of vertical cuts was set to 2 m. The algorithm was provided with vertical cuts as input to complete the modeling process, which resulted in the re-modeling of the quarry face. Figure 6 displays the graphical output of "Model 1" along with the vertical cuts and the grading of the formed blocks.

The results related to the calculation of the main outputs of the algorithm, including cutting levels, the value of blocks, and BCSdbBV are shown in Table 3. Also, the specifications of the blocks tracked in the model and their grading are presented in Table 4.

**Table 3:** Results of calculation of BCSdbBV in “Model 1” with traditional cutting pattern.

BCS	BV	BCSdbBV
229	32.08	7.14

**Table 4:** Specifications of formed blocks in “Model 1” with traditional cutting pattern.

Row	Block volume (m^3^)	Block shape factor	Useful block volume	Block class	Block value
1	16.33	0.94	15.32	'Good'	3.83
2	16.28	0.95	15.51	'Good'	3.88
3	16.2	0.98	15.82	'Good'	3.96
4	16.05	0.98	15.72	'Good'	3.93
5	15.89	0.96	15.28	'Good'	3.82
6	12.48	0.89	11.07	'Good'	2.77
7	12.17	0.93	11.32	'Good'	2.83
8	11.06	0.93	10.26	'Good'	2.57
9	9.08	0.87	7.89	'Medium'	0.79
10	8.93	0.87	7.74	'Medium'	0.77
11	6.96	0.82	5.74	'Medium'	0.57
12	6.74	0.95	6.4	'Medium'	0.64
13	5.84	0.86	5	'Medium'	0.5
14	5.84	0.96	5.58	'Medium'	0.56
15	3.9	0.88	3.42	'Medium'	0.34
16	3.29	0.99	3.24	'Medium'	0.32
17	2.14	0.68	1.46	'Waste'	0
18	2.09	0.84	1.75	'Waste'	0
19	1.95	0.86	1.69	'Waste'	0
20	1.79	0.86	1.53	'Waste'	0
21	1.72	0.8	1.37	'Waste'	0
22	1.62	0.69	1.12	'Waste'	0
23	1.57	0.69	1.09	'Waste'	0
24	0.1	0.8	0.08	'Waste'	0
25	0.02	0.7	0.01	'Waste'	0
26	0.01	0.7	0.01	'Waste'	0
Sum	180	–	165.44	–	32.08

According to the calculations in Table 3, the BCSdbBV value suggests that 7.14 m^2^ of cutting surfaces must be cut for each valuable block unit. Additionally, based on the number and value of the blocks, the overall efficiency of the model is estimated to be around 18%, assuming the given conditions.

#### Model 2

To implement and evaluate the developed algorithm, another hypothetical model with different discontinuities specifications is considered "Model 2". Dimensions of the model range, block grading specifications, and optimization parameters are considered Similar to "Model 1" specifications. The discontinuities specifications of "Model 2" are shown in Table 5.

**Table 5:** Specifications of discontinuities in “Model 2”.

Joint number	Joint type	Dip (degree)	Dip direction (degree)	Coordinates of a point			Radius
				X	Y	Z	
1	Complete	45	180	2	0	0	–
2	Complete	45	180	2	2	0	–
3	Incomplete^a^	30	0	1	9	4.5	5.2
4	Complete	70	180	2	9	4.5	–
5	Complete	30	0	2	10	0	–
6	Complete	70	180	2	12.5	0	–
7	Complete	65	180	2	14.5	0	–
8	Complete	60	0	2	20	0	–

^a^A joint that has limited dimensions

**Figure 7:**
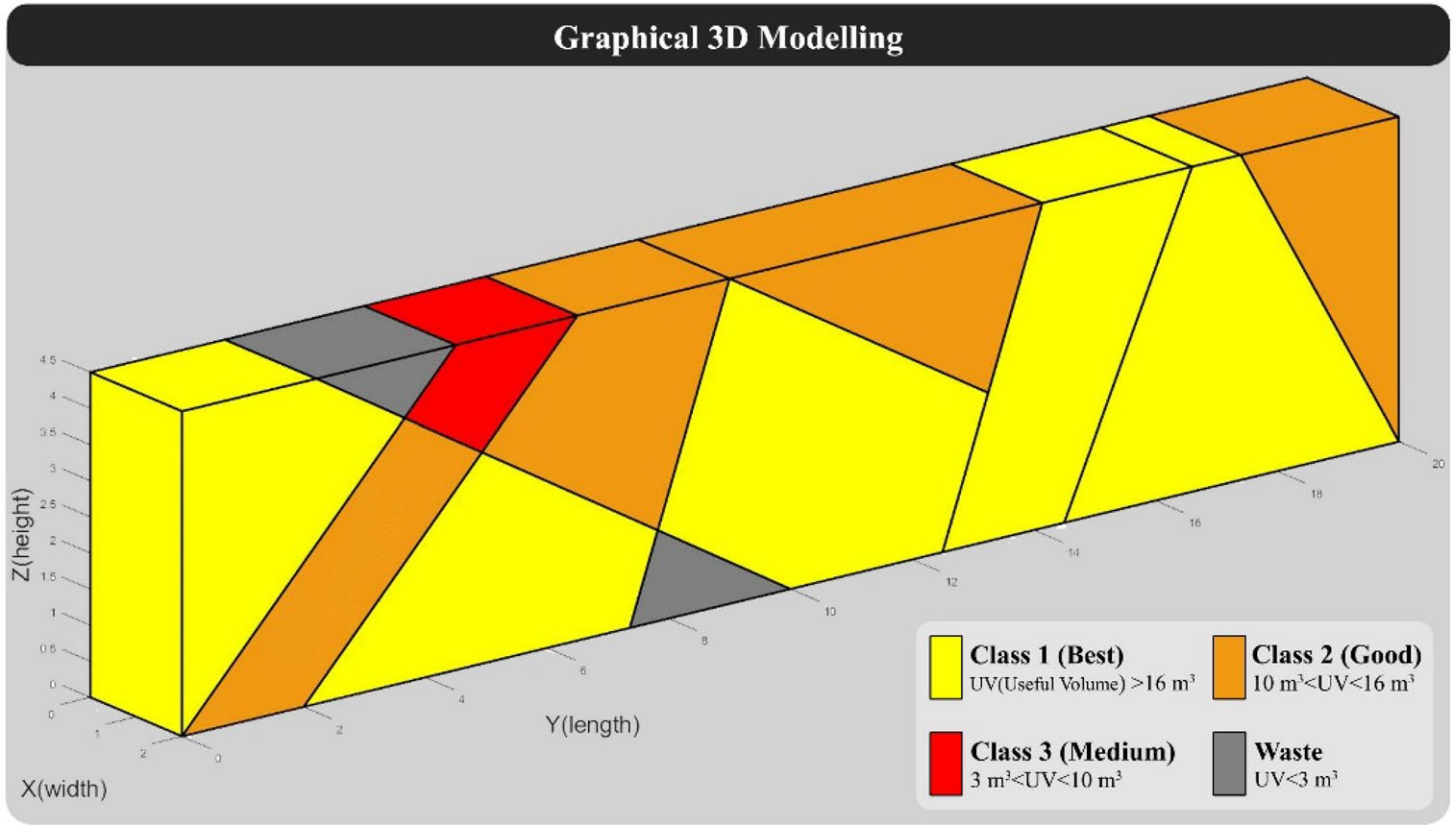
3D modeling of “Model 2” without considering vertical cuts.

Based on the specifications of "Model 2", the programmed algorithm was executed on it, in the following displayed the results. Figure 7 shows the 3D modeling of "Model 2" without taking into account the vertical cuts of the quarry face.

**Figure 8:**
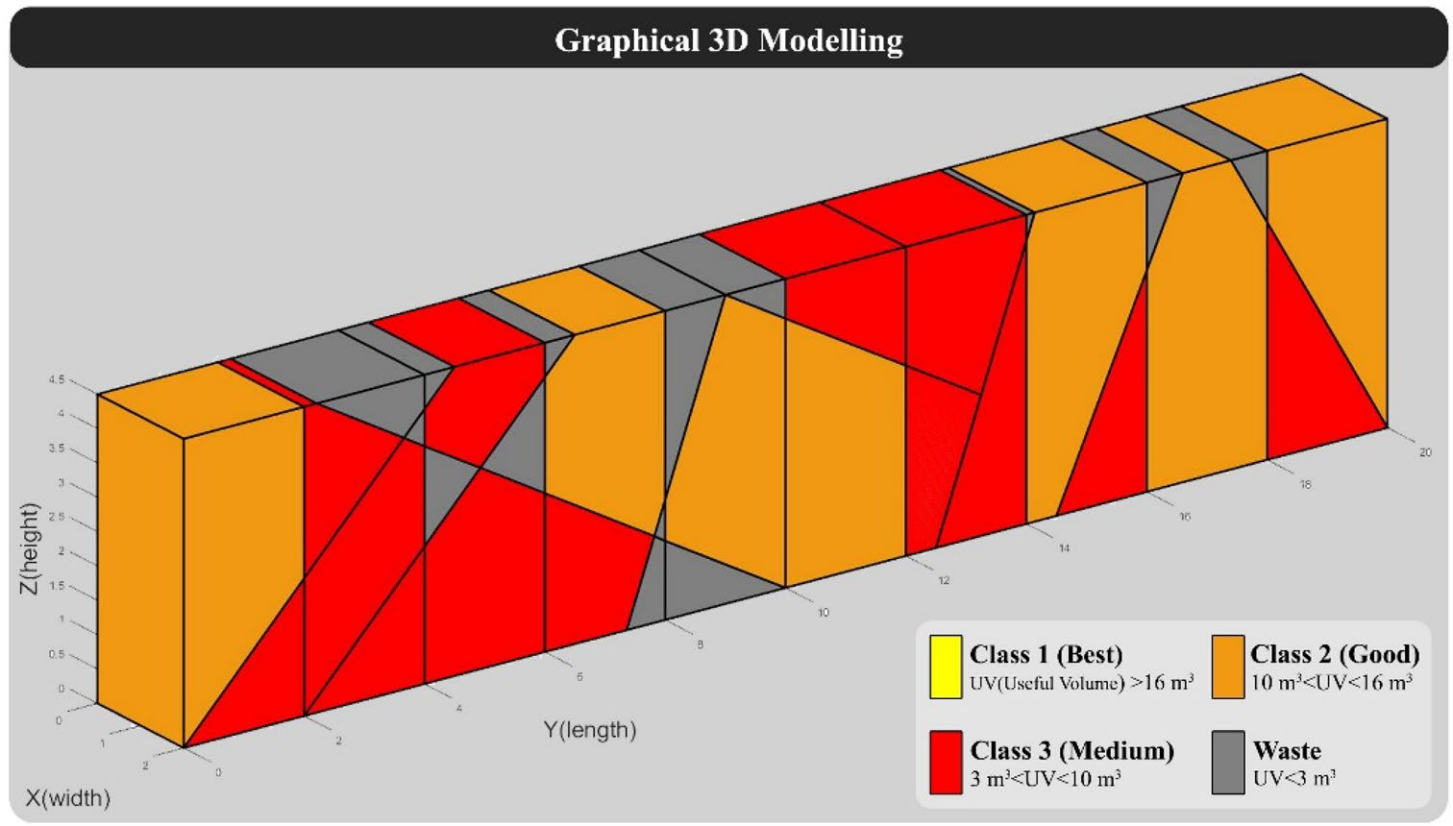
3D modeling of “Model 2” with traditional cutting pattern.

The distance between vertical cuts in this model was set at 2 m. The hypothetical quarry was then re-modeled based on the characteristics of discontinuities and vertical cuts. Figure 8 shows the graphical output of "Model 2", which includes the vertical cuts and the grading of the formed blocks.

Table 6 displays the main outputs of the algorithm, and Table 7 presents the specifications and grading of tracked blocks in the model.

**Table 6:** Results of calculation of BCSdbBV in "Model 2" with traditional cutting pattern.

BCS	BV	BCSdbBV
229	28.69	7.98

**Table 7:** Specifications of blocks formed in Model 2 with traditional cutting pattern.

row	Block volume (m^3^)	Block shape factor	Useful block volume	Block class	Block value
1	16.61	0.93	15.46	'Good'	3.86
2	14	0.99	13.83	'Good'	3.46
3	13.38	0.95	12.71	'Good'	3.18
4	13.12	0.93	12.18	'Good'	3.05
5	12.37	0.97	11.98	'Good'	2.99
6	11.08	0.93	10.33	'Good'	2.58
7	10.71	0.98	10.51	'Good'	2.63
8	10.19	0.95	9.68	'Medium'	0.97
9	7.82	0.95	7.4	'Medium'	0.74
10	7.35	0.95	6.98	'Medium'	0.7
11	6.92	0.9	6.23	'Medium'	0.62
12	6.18	0.82	5.09	'Medium'	0.51
13	5.92	0.95	5.62	'Medium'	0.56
14	4.83	0.86	4.14	'Medium'	0.41
15	4.62	0.95	4.39	'Medium'	0.44
16	4.49	0.95	4.28	'Medium'	0.43
17	4.39	0.95	4.16	'Medium'	0.42
18	4.33	0.96	4.14	'Medium'	0.41
19	4	0.91	3.66	'Medium'	0.37
20	4	0.91	3.66	'Medium'	0.37
21	2.75	0.78	2.15	'Waste'	0
22	2.3	0.84	1.94	'Waste'	0
23	1.81	0.89	1.61	'Waste'	0
24	1.67	0.85	1.43	'Waste'	0
25	1.36	0.87	1.18	'Waste'	0
26	1.01	0.84	0.85	'Waste'	0
27	0.77	0.73	0.56	'Waste'	0
28	0.62	0.74	0.46	'Waste'	0
29	0.58	0.74	0.43	'Waste'	0
30	0.25	0.69	0.17	'Waste'	0
31	0.25	0.69	0.17	'Waste'	0
32	0.18	0.69	0.13	'Waste'	0
33	0.11	0.52	0.06	'Waste'	0
34	0.05	0.48	0.03	'Waste'	0
Sum	180	–	167.59	–	28.69

BCSdbBV value in Table 7 indicates that 7.98 m^2^ of cutting surfaces should be cut for each unit of valuable block. Also, according to the number of blocks and their value, the overall efficiency of the model according to its assumptions is about 16%.

## Cutting pattern optimization

**Figure 9:**
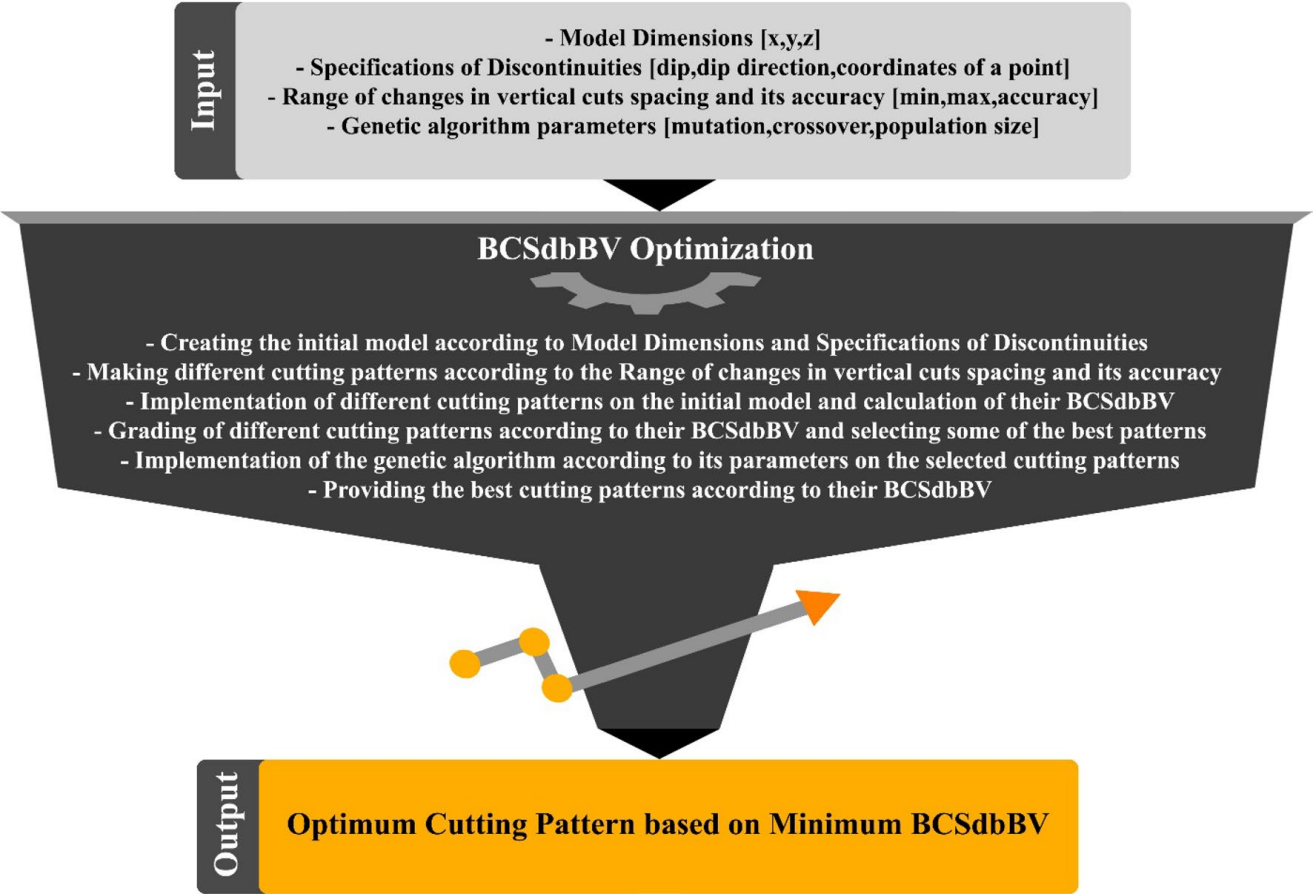
General process of the programmed optimization algorithm.

Providing an optimal cutting pattern in the quarry face of dimension stones quarries is essential to increase production efficiency. The spacing of vertical cuts in dimension stone quarries is usually considered a fixed value regardless of the geological conditions of the quarry face. Using a fixed cutting pattern in the quarry face, regardless of the change in the geological conditions of different quarries, can increase operating costs (cost of water, energy, and cutting tools) and decrease the value of produced blocks (increase in production wastes) and finally Reduce operational efficiency. On the other hand, practical testing of all different cutting patterns in quarries is impossible. All these points make it necessary to provide an algorithm for optimizing the cutting pattern in dimension stone quarry faces. In the previous section, the implementation of the BCSdbBV calculation algorithm based on the discontinuity specifications and the spacing of the vertical cuts was fully expressed. To optimize the block cutting pattern in the quarry face, a new optimization algorithm was programmed in MATLAB based on a genetic algorithm, the main purpose of which is to provide the optimal cutting pattern based on the minimization value of BCSdbBV. The general process of the programmed optimization algorithm is shown in Fig. 9.

As shown in Fig. 9, the optimization algorithm receives the dimensions of the model range and the specifications of discontinuities as inputs, and after performing the optimization operation based on the desired optimization parameters, provides the optimal quarry face cutting pattern based on minimum BCSdbBV as the main output and provides some of the best cutting patterns as a secondary output if needed.

## Results and discussion

At the start of the optimization algorithm evaluation process, model 1 (displayed in Fig. 6) was identified as the target quarry face. Extracting blocks from a quarry face involves cutting and drilling vertical and horizontal holes, which can be quite costly. Considering these costs, a spacing of less than 1.5 m between vertical cuts is not economical. Therefore, the optimization algorithm uses a minimum distance of 1.5 m between vertical cuts. Additionally, if the distance between vertical cuts is more than 3 m, the resulting blocks may be too heavy to transport due to weight limits. Hence, the optimization algorithm uses a maximum distance of 3 m between vertical cuts. This ensures that only blocks within a transportable weight range are produced.

Finally, according to operational limits, the accuracy of the spacing of vertical cuts was considered 0.5 m (the spacing of vertical cuts can be 1.5, 2, 2.5, or 3 m). also, the simulation and optimization process were time-consuming, so the initial population number was set to 100. After multiple tests and evaluations, the crossover and mutation parameters were set to 0.8 and 0.02, respectively^35^. Based on the given assumptions, the results of the optimization algorithm are presented in Table 8, which shows the optimal cutting pattern in "model 1" based on the minimum BCSdbBV.

**Table 8:** Optimal cutting pattern in “Model 1” based on the minimum BCSdbBV.

Vertical cutting points (meters)	
1.5–4.5–7.5–10–12.5–14–17	

**Figure 10:**
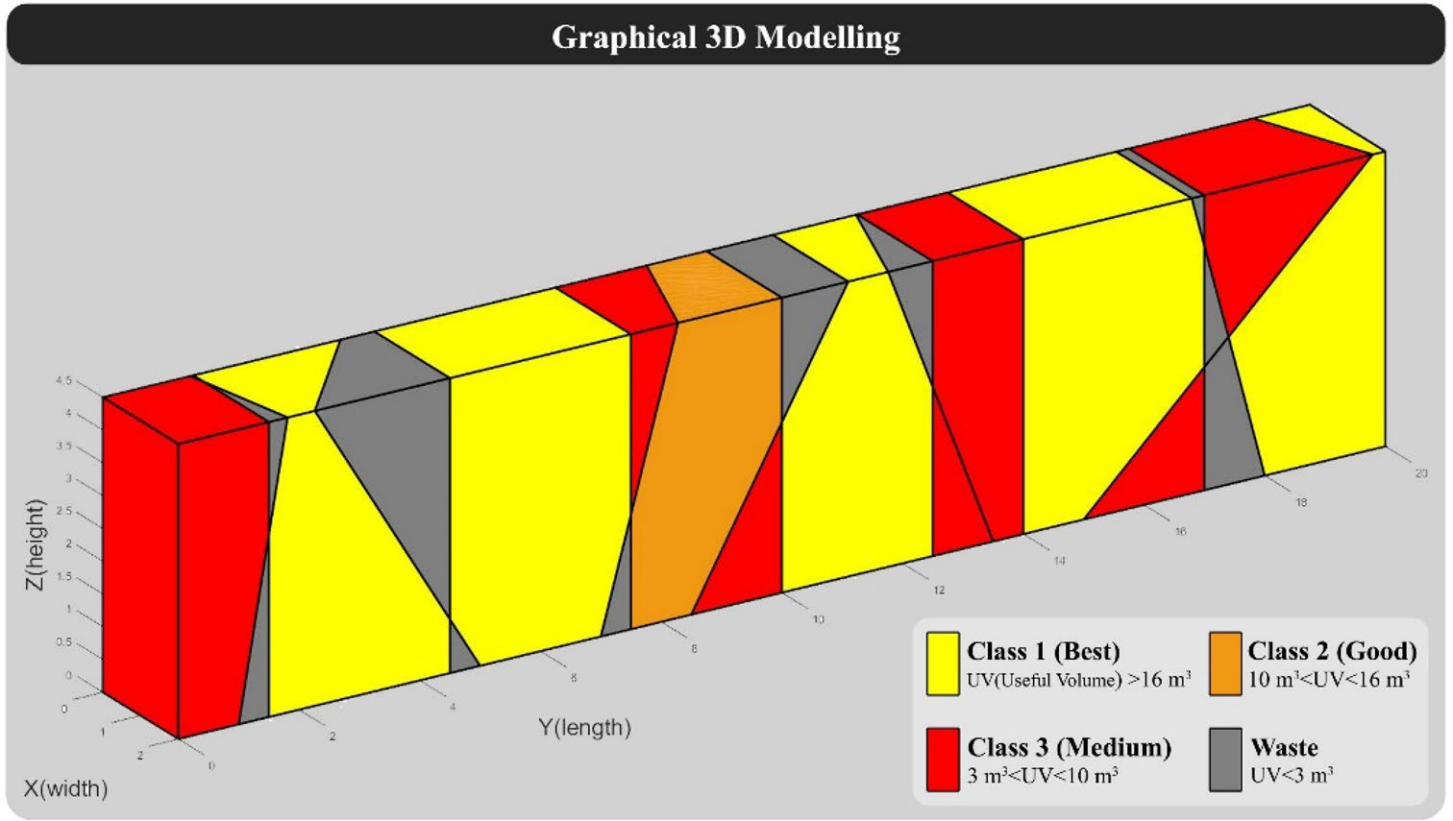
3D modeling of “Model 1” with optimal cutting pattern.

The graphical output of “Model 1” after implementing the optimal cutting pattern (Table 8) is shown in Fig. 10.

Also, the numerical outputs of using the optimal cutting pattern for “Model 1” are shown in Tables 9 and 10.

**Table 9:** Results of calculation of BCSdbBV in "Model 1" with optimal cutting pattern.

BCS	BV	BCSdbBV
211	107.57	1.96

**Table 10:** Specifications of blocks formed in “Model 1” with optimal cutting pattern.

Row	Block volume (m^3^)	Block shape factor	Useful block volume	Block class	Block value
1	23.47	0.94	22.08	'Best'	22.08
2	23.24	0.93	21.72	'Best'	21.72
3	21.53	0.96	20.73	'Best'	20.73
4	19.93	0.94	18.82	'Best'	18.82
5	18.19	0.95	17.28	'Best'	17.28
6	13.85	0.9	12.49	'Good'	3.12
7	10.89	0.9	9.76	'Medium'	0.98
8	8.6	0.88	7.54	'Medium'	0.75
9	5.29	0.92	4.85	'Medium'	0.49
10	5.08	0.95	4.81	'Medium'	0.48
11	4.9	0.81	3.99	'Medium'	0.4
12	4.75	0.76	3.62	'Medium'	0.36
13	3.9	0.88	3.42	'Medium'	0.34
14	3.61	0.82	2.95	'Waste'	0
15	3.33	0.82	2.72	'Waste'	0
16	3.24	0.89	2.87	'Waste'	0
17	2.62	0.63	1.64	'Waste'	0
18	2.09	0.84	1.75	'Waste'	0
19	0.5	0.58	0.29	'Waste'	0
20	0.49	0.7	0.34	'Waste'	0
21	0.2	0.66	0.13	'Waste'	0
22	0.16	0.51	0.08	'Waste'	0
23	0.15	0.52	0.08	'Waste'	0
24	0.01	0.7	0.01	'Waste'	0
Sum	180	–	164.01	–	107.57

**Figure 11:**
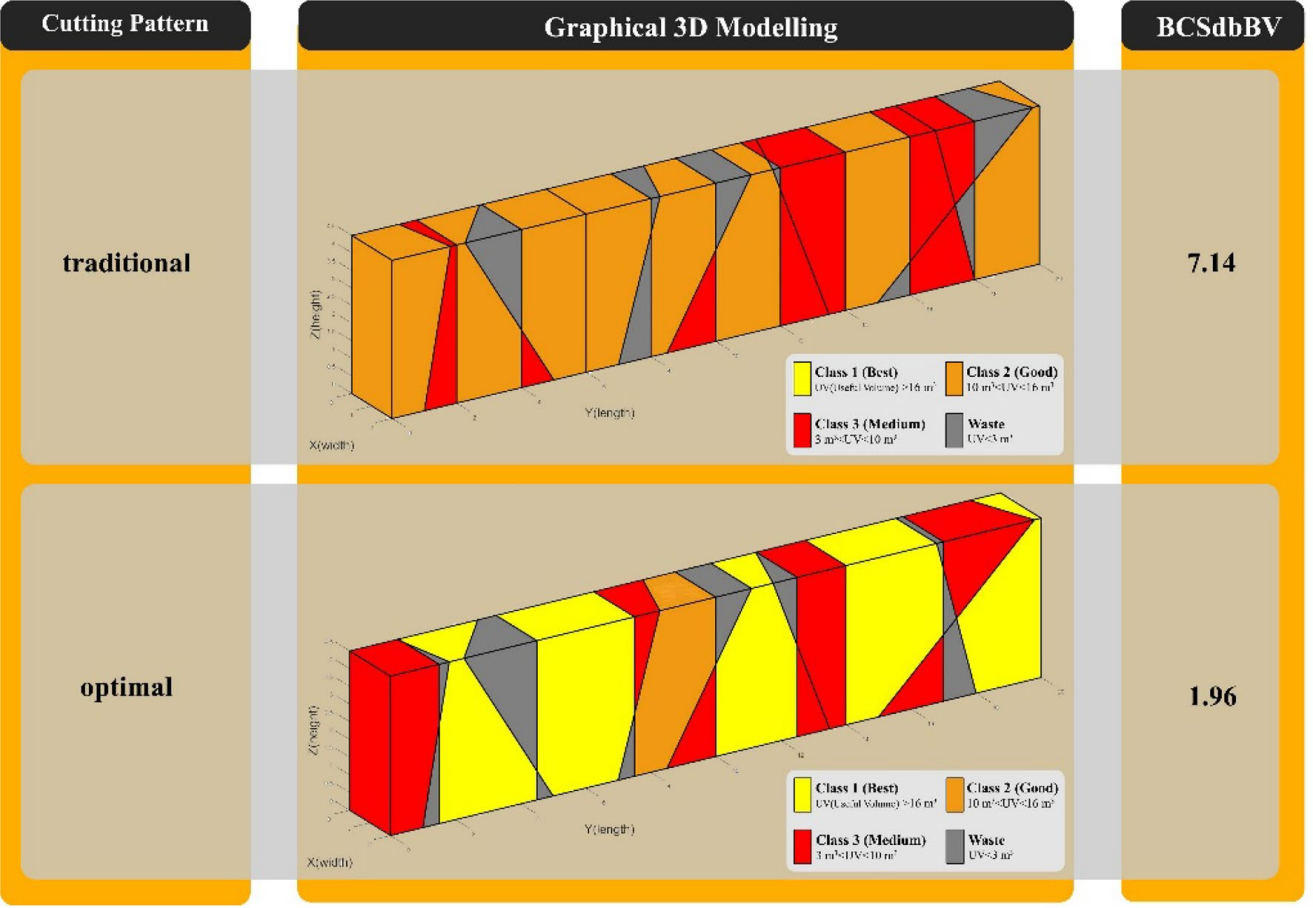
Comparison of “Model” 1 in two modes of traditional and optimal cutting patterns.

**Figure 12:**
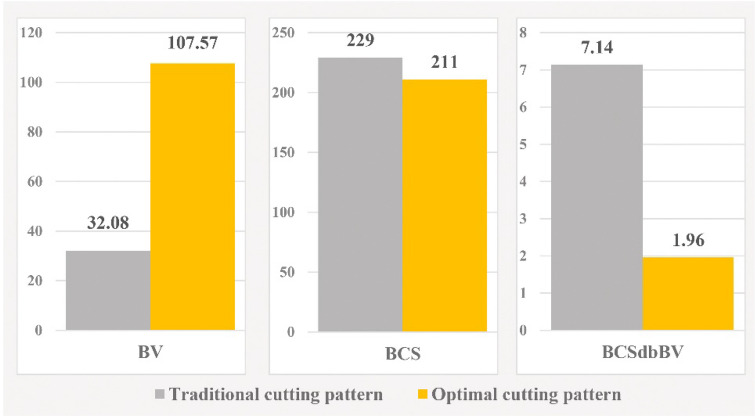
Comparison graph of outputs of “Model 1” in two modes of traditional and optimal cutting patterns.

In Fig. 11, "Model 1" is compared in the two modes of implementation of the traditional and optimal cutting patterns. Also, to better demonstrate the performance of the new optimization algorithm, a comparison of outputs in the two modes of implementation of the traditional cutting pattern and the optimal cutting pattern is shown in Fig. 12.

**Figure 13:**
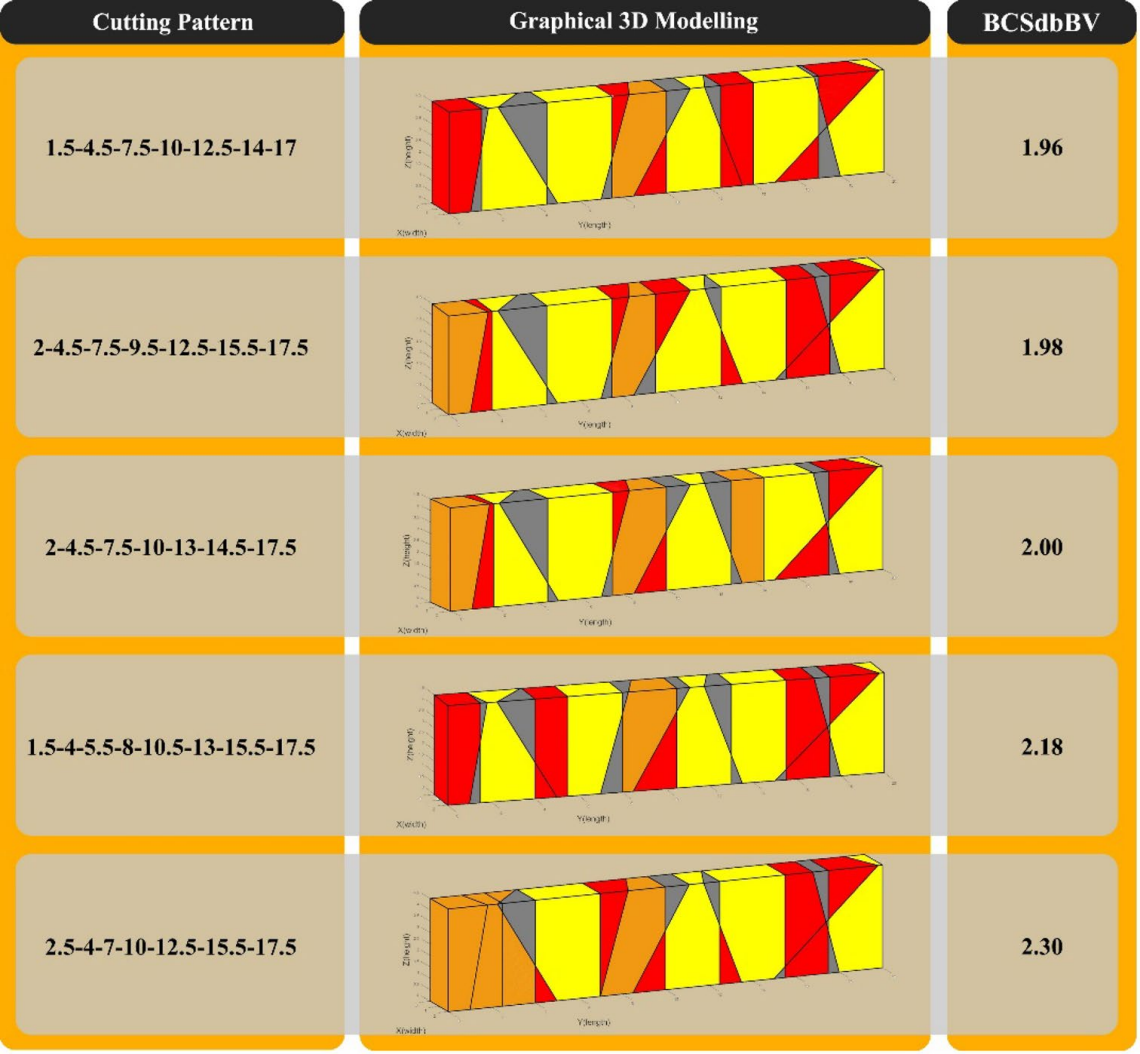
The first five cases of the best cutting patterns proposed by the optimization algorithm for "Model 1".

The best cutting patterns proposed by the programmed optimization algorithm, with calculated BCSdbBV for each pattern, are shown in Fig. 13.

The optimization algorithm evaluation process continued with consideration of the "Model 2" (displayed in Fig. 7) and the implementation of the algorithm on it. In the following, the results of the optimization algorithm are presented. Table 11 displays the optimal cutting pattern for "Model 2" based on the minimum BCSdbBV.

**Table 11:** Optimal cutting pattern in "Model 2" based on the minimum BCSdbBV.

Vertical Cutting points (meters)	
3–5.5–8–10–13–16–18.5	

**Figure 14:**
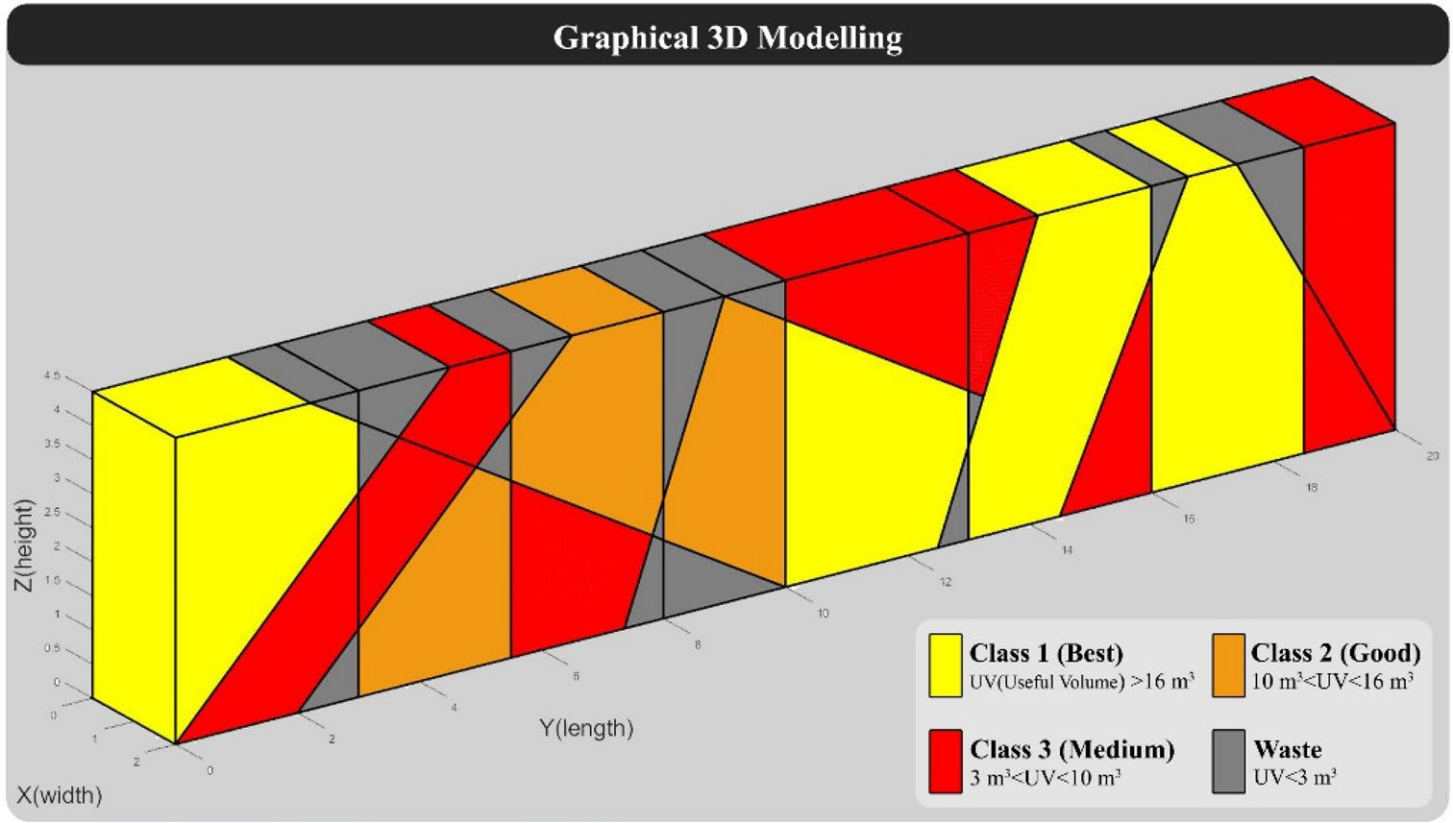
3D modeling of “Model 2” with optimal cutting pattern.

The graphical output of "Model 2" after implementing the optimal cutting pattern (Table 11) is shown in Fig. 14.

Also, the numerical output of the algorithm if using the optimal cutting pattern for "Model 2" is shown in Tables 12 and 13.

**Table 12:** Results of calculation of BCSdbBV in "Model 2" with optimal cutting pattern.

BCS	BV	BCSdbBV
211	82.22	2.56

**Table 13:** Specifications of formed blocks in “Model 2” with optimal cutting pattern.

Row	Block volume (m^3^)	Block shape factor	Useful block volume	Block class	Block value
1	19.64	0.94	18.5	'Best'	18.5
2	18.6	0.88	16.36	'Best'	16.36
3	17.65	0.95	16.76	'Best'	16.76
4	17.64	0.96	16.91	'Best'	16.91
5	12.37	0.97	11.98	'Good'	2.99
6	12	0.98	11.73	'Good'	2.93
7	10.74	0.95	10.2	'Good'	2.55
8	9.6	0.92	8.85	'Medium'	0.88
9	8.67	0.99	8.56	'Medium'	0.86
10	8.38	0.95	7.96	'Medium'	0.8
11	8	0.94	7.51	'Medium'	0.75
12	5.18	0.99	5.11	'Medium'	0.51
13	4.83	0.86	4.14	'Medium'	0.41
14	3.9	0.88	3.42	'Medium'	0.34
15	3.82	0.96	3.66	'Medium'	0.37
16	3.37	0.9	3.02	'Medium'	0.3
17	2.75	0.78	2.15	'Waste'	0
18	2.3	0.84	1.94	'Waste'	0
19	2.09	0.84	1.75	'Waste'	0
20	1.56	0.92	1.44	'Waste'	0
21	1.01	0.84	0.85	'Waste'	0
22	1	0.82	0.82	'Waste'	0
23	1	0.82	0.82	'Waste'	0
24	0.77	0.73	0.56	'Waste'	0
25	0.69	0.81	0.56	'Waste'	0
26	0.69	0.68	0.46	'Waste'	0
27	0.58	0.74	0.43	'Waste'	0
28	0.51	0.78	0.4	'Waste'	0
29	0.36	0.7	0.25	'Waste'	0
30	0.21	0.58	0.12	'Waste'	0
31	0.11	0.52	0.06	'Waste'	0
Sum	180	–	167.25	–	82.22

**Figure 15:**
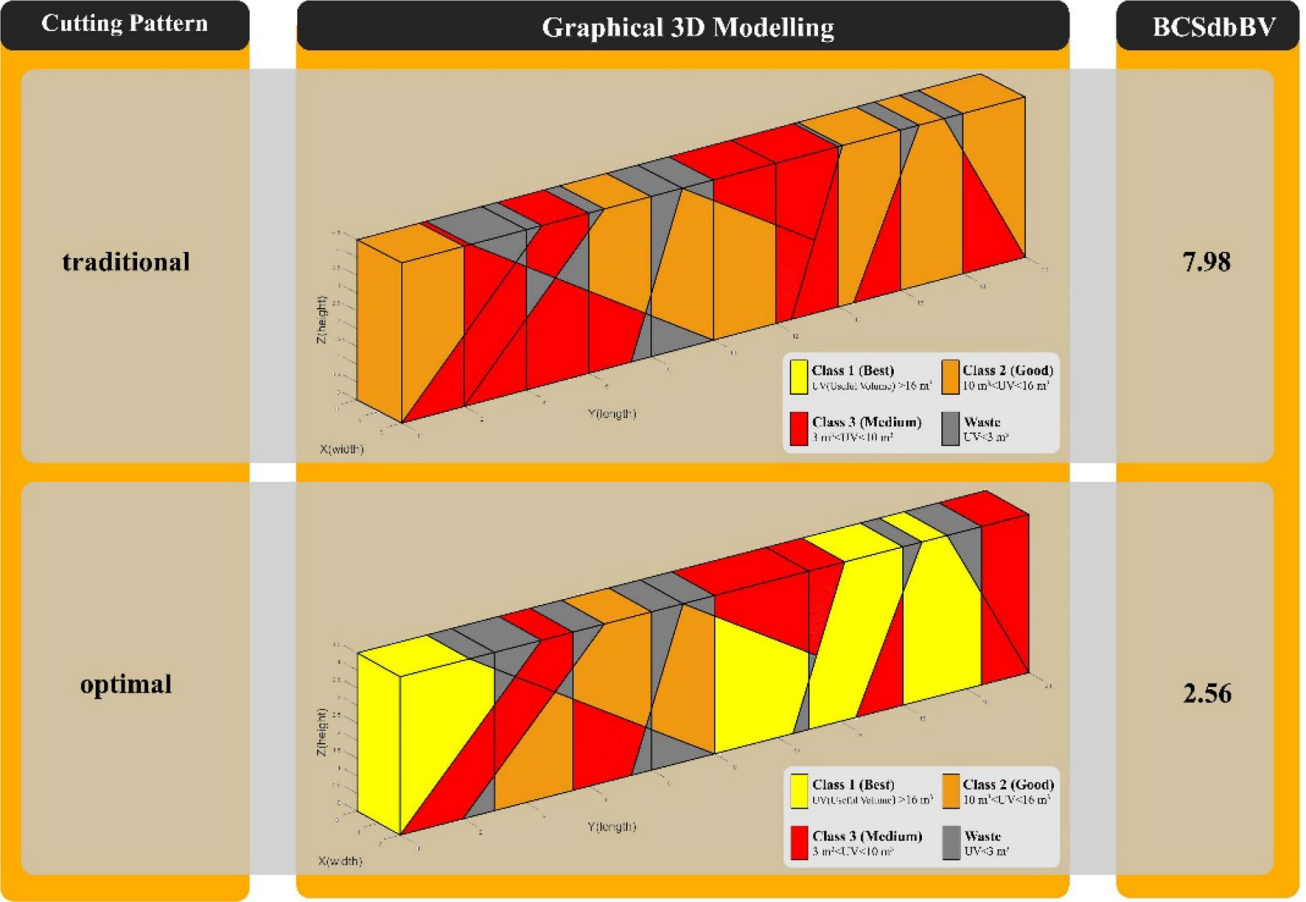
Comparison of “Model 2” in two modes of traditional and optimal cutting patterns.

**Figure 16:**
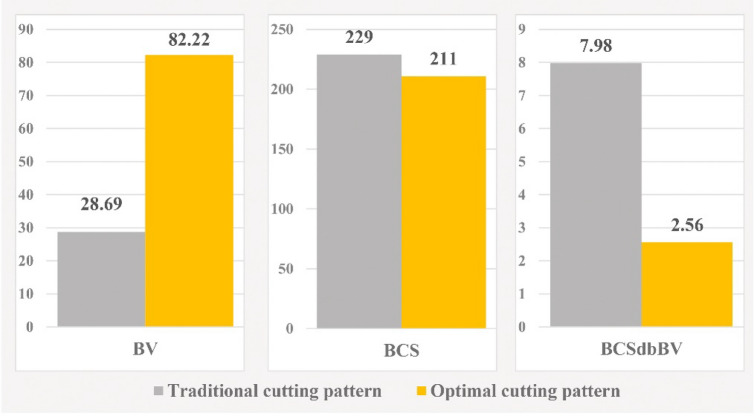
Comparison graph of outputs of “Model 2” in two modes of traditional and optimal cutting patterns.

In Fig. 15, "Model 2" is compared in the two modes of implementation of the traditional and optimal cutting patterns. Also, as in "Model 1", to better demonstrate the performance of the optimization algorithm, a comparison of outputs in the two modes of implementation of the traditional cutting pattern and the optimal cutting pattern is shown in Fig. 16.

**Figure 17:**
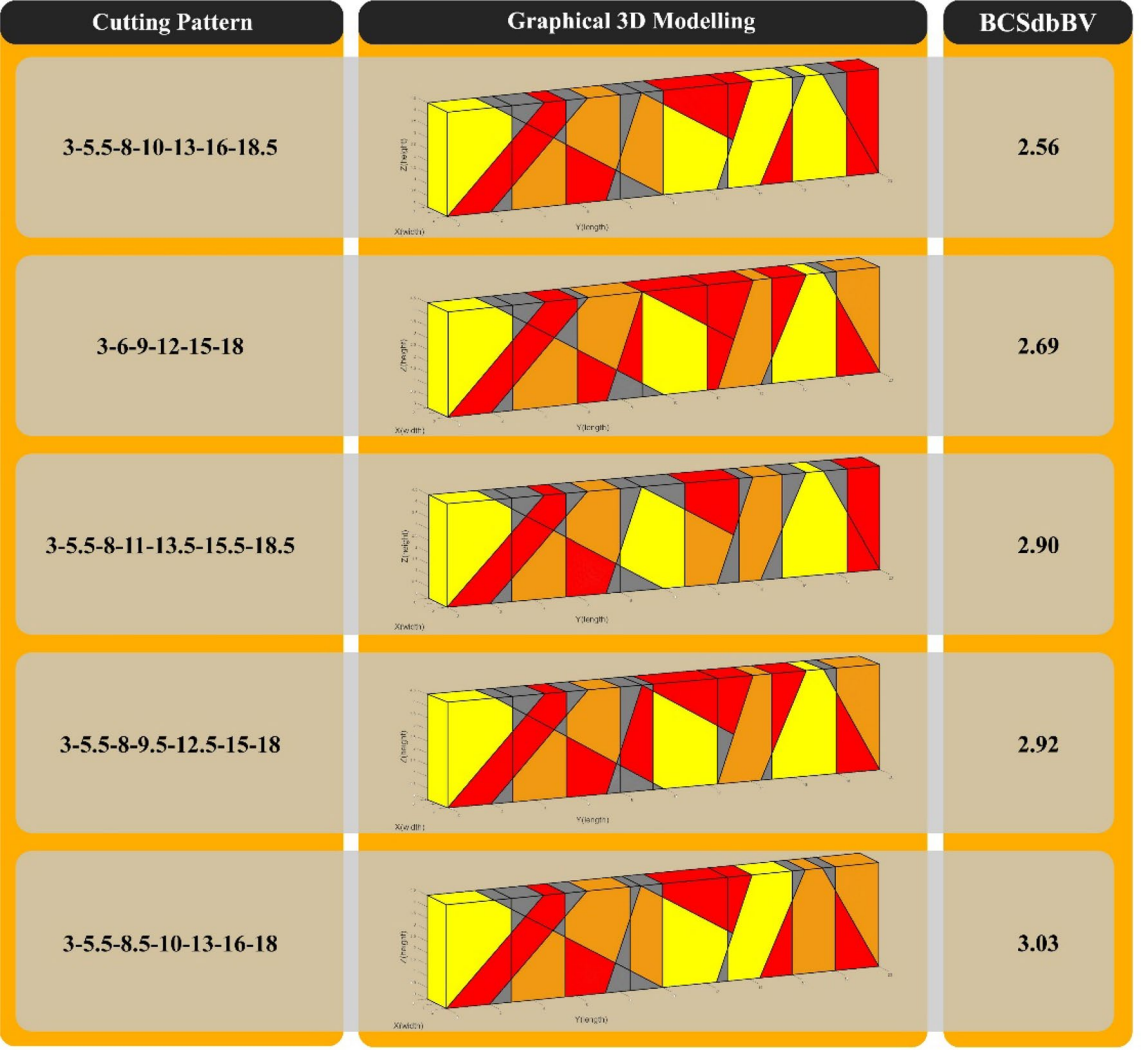
The first five cases of the best cutting patterns proposed by the optimization algorithm for "Model 2".

The best cutting patterns proposed by the programmed optimization algorithm, with calculated BCSdbBV for each pattern, are shown in Fig. 17.

Based on the results presented, it can be inferred that implementing the optimization algorithm can be advantageous for the economic and environmental optimization of dimension stone quarries. The hypothetical model used in this study was based on an actual quarry face. Moreover, the suggested cutting patterns are fully practicable, and all operational considerations have been considered.

In "Model 1", according to Figs. 11 and 12, the use of optimal cutting patterns has a significant impact on the parameters affecting the optimization of dimension stone quarrying. The "BV" parameter has increased to 107.57 units from 32.08 units (a 235% increase), resulting in a higher number of valuable blocks and a lower volume of waste blocks. Additionally, the "BCS" parameter has reduced from 229 to 211 m^2^ (an 8% reduction), leading to reduced operating costs of block extraction, such as water, energy, and cutting tool consumption. This reduction also saves time by decreasing the block cutting time. Finally, the "BCSdbBV" parameter has decreased from 7.14 units to 1.96 units (a 72% reduction), indicating that the cutting surfaces per valuable block unit have reached the initial value of about 27%. In case there are any limitations in implementing the optimal cutting pattern shown in Fig. 10, the cutting patterns illustrated in Fig. 13 can also be utilized. Based on the BCSdbBV value of cutting patterns presented in Fig. 13, implementing any of these patterns can lead to more optimal results compared to the traditional cutting pattern.

In "Model 2", as shown in Figs. 15 and 16, the value of the "BV" parameter increased by about 186%, from 28.69 units to 82.22 units. This increase led to a rise in the number of valuable blocks and a decrease in the volume of waste blocks. Moreover, the "BCS" parameter was reduced by 8%, resulting in a reduction in operating costs and cutting time. Lastly, the "BCSdbBV" parameter also decreased from 7.98 units to 2.56 units (about 68% reduction), indicating that the cutting surfaces per valuable block unit have reached their initial value of about 32%. to achieve better results than the traditional cutting pattern in "Model 1", the cutting patterns proposed in Fig. 17 can also be used.

Although the algorithm presented in this paper has demonstrated favorable results, it can still achieve better outcomes. As mentioned earlier, due to time constraints during the optimization process, the genetic algorithm's initial population size was limited to 100, and the accuracy of the vertical cuts was set to 0.5 m. However, if more powerful computers are utilized during the optimization process and the time constraints are relaxed, it's possible to increase the initial population size and reduce the accuracy of the vertical cut spacing (for instance, 0.25 m). By doing so, we can arrive at more optimal cutting patterns.

## Conclusions

The dimension stones industry has enormous economic potential, However, this industry faces several challenges such as improper operation, significant resource loss, and waste production, resulting in low efficiency and high production costs. According to data published in 2021, approximately 51% of the total gross quarrying in quarries will be converted into waste. This increase in waste production and resource loss leads to the misallocation of energy, water, and cutting tools that should be used for production, thus increasing costs, and reducing efficiency. In recent years, many studies have been conducted to optimize dimension stone quarries, which have yielded positive results. However, these studies tend to overlook other critical parameters, such as energy, water, and cutting tools consumption, that are essential for the environmental and economic optimization of dimension stone quarrying. To optimize dimension stone quarrying, producers should focus not only on producing economic blocks with large dimensions but also on parameters that affect operational costs, such as energy consumption.

In this study, an optimization algorithm was developed for dimension stone quarrying to consider all economic and environmental parameters affecting the optimization process. The algorithm takes the characteristics of the quarry (including the dimensions of the quarry face and the characteristics of the discontinuities) as input, and after performing the optimization process, provides the optimal cutting pattern of the quarry face. The goal of this algorithm is to maximize the value of the extracted blocks (decreasing the amount of waste) and minimize cutting surfaces (reducing energy, water, and cutting tools consumption), it achieves this goal by minimizing the BCSdbBV. To evaluate the developed algorithm, two hypothetical models, "Model 1" and "Model 2," were created using real data. After running the optimization algorithm on the two models, the following results were obtained:

- In model 1, if the traditional cutting pattern is used, the value of BCSdbBV will be 7.14 square meters for each unit of valuable block. However, if the optimal cutting pattern recommended by the optimization algorithm is implemented, this amount will significantly reduce to 1.96 m^2^ which is about 72% less.
- In Model 2, when using the traditional cutting pattern, the value of BCSdbBV is 7.98 square meters per unit of valuable block. However, if you implement the optimal cutting pattern provided by the optimization algorithm, this value will decrease to 2.56 m^2^, which is a significant 67% reduction.
- The optimal cutting pattern based on discontinuities specification and minimizing BCSdbBV can reduce cutting surfaces and increase valuable blocks, resulting in lower energy, water, and tool consumption, and reduced waste production.

In general, quarry optimization based on the BCSdbBV parameter can effectively optimize most of the operational parameters, reduce resource losses, and significantly increase production efficiency. The presented optimization algorithm considers all operational constraints in dimension stone quarrying. Since diamond wire machines are commonly used for dimension stone quarrying, changing the spacing of vertical cuts is operationally feasible. As a result, an optimal cutting pattern with variable spacing of vertical cuts can be used instead of the traditional pattern with fixed spacing. Implementation of this method in dimension stone quarries requires an understanding of the discontinuities conditions in the quarry face and can be achieved through a simple geological survey. Ultimately, this algorithm can serve as a means for environmentally and economically optimizing dimension stone quarries.

## Data Availability

All data generated or analyzed during this study are included in this published article (and its Supplementary Information files).
